# Diversity and Distribution of Prokaryotes within a Shallow-Water Pockmark Field

**DOI:** 10.3389/fmicb.2016.00941

**Published:** 2016-06-17

**Authors:** Donato Giovannelli, Giuseppe d'Errico, Federica Fiorentino, Daniele Fattorini, Francesco Regoli, Lorenzo Angeletti, Tatjana Bakran-Petricioli, Costantino Vetriani, Mustafa Yücel, Marco Taviani, Elena Manini

**Affiliations:** ^1^Institute of Marine Sciences, National Research CouncilAncona, Italy; ^2^Institute of Earth, Ocean and Atmospheric Science, Rutgers, The State University of New JerseyNew Brunswick, NJ, USA; ^3^Program in Interdisciplinary Studies, Institute of Advanced StudiesPrinceton, NJ, USA; ^4^Earth-Life Science Institute, Tokyo Institute of TechnologyTokyo, Japan; ^5^Department for Life and Environmental Science, Polytechnic University of MarcheAncona, Italy; ^6^Institute of Marine Sciences, National Research CouncilBologna, Italy; ^7^Department of Biology, Faculty of Science, University of ZagrebZagreb, Croatia; ^8^Department of Biochemistry and Microbiology, Rutgers, The State University of New JerseyNew Brunswick, NJ, USA; ^9^Institute of Marine Sciences, Middle East Technical UniversityMersin, Turkey; ^10^Department of Biology, Woods Hole Oceanographic InstitutionWoods Hole, MA, USA

**Keywords:** pockmarks, cold seeps, hydrocarbons, prokaryotic diversity, bacteria, archaea, hydrocarbon degradation, microbial diversity

## Abstract

Pockmarks are crater-like depression on the seafloor associated with hydrocarbon ascent through muddy sediments in continental shelves around the world. In this study, we examine the diversity and distribution of benthic microbial communities at shallow-water pockmarks adjacent to the Middle Adriatic Ridge. We integrate microbial diversity data with characterization of local hydrocarbons concentrations and sediment geochemistry. Our results suggest these pockmarks are enriched in sedimentary hydrocarbons, and host a microbial community dominated by *Bacteria*, even in deeper sediment layers. Pockmark sediments showed higher prokaryotic abundance and biomass than surrounding sediments, potentially due to the increased availability of organic matter and higher concentrations of hydrocarbons linked to pockmark activity. Prokaryotic diversity analyses showed that the microbial communities of these shallow-water pockmarks are unique, and comprised phylotypes associated with the cycling of sulfur and nitrate compounds, as well as numerous know hydrocarbon degraders. Altogether, this study suggests that shallow-water pockmark habitats enhance the diversity of the benthic prokaryotic biosphere by providing specialized environmental niches.

## Introduction

Pockmarks are a type of cold seeps characterized by seafloor depression induced by fluids or hydrocarbon ascent through muddy sediments (Judd and Hovland, 2007). Natural gas and fluid migration to the surface create a mass defect under the seafloor generating a gravity collapse and the typical crater-like depressions (Judd and Hovland, 2007). Pockmarks generally occur in fine-grained sediments as cone-shaped circular depressions ranging from few meters to >500 m in diameter and from 1 to 80 m in depth. Pockmarks are widespread features along passive and active continental margins and they are often present in clusters varying from few to hundreds per km^2^ (Pilcher and Argent, 2007; Forwick et al., 2009). Due to their abundance and distribution, which includes shallow-water locations, the contribution of pockmarks to global methane and hydrocarbon release could be enormous (Fleischer et al., 2001). Methane, despite its low abundance in the atmosphere, is a powerful greenhouse gas and has been recognized as one of the forcing factors for rapid climate change (Manne and Richels, 2001; Watson, 2001). Its release from the ocean floor can influence global warming, modify oceanic redox conditions and affect the global carbon cycle (Hinrichs and Boetius, 2003; Judd and Hovland, 2007; Reeburgh, 2007). Seepage of methane and other hydrocarbons can sustain locally dense populations of metazoans supported by aerobic methane and/or sulfide oxidizing prokaryotes (Boetius et al., 2000; Olu-Le Roy et al., 2004; Judd and Hovland, 2007; Taviani, 2011). While the microbial communities associated with the degradation of methane have been widely investigated in seep environments (Knittel and Boetius, 2009; Ruff et al., 2015), microbial communities associated with the natural seepage of heavier hydrocarbons have received little attention.

Previous studies carried out in the North Sea, Atlantic Ocean, and Mediterranean Sea revealed that pockmarks may represent hot-spot of biodiversity, both for the microbial community and metazoans (Olu-Le Roy et al., 2004; Bouloubassi et al., 2009; Cambon-Bonavita et al., 2009; Merkel et al., 2010; Pimenov et al., 2010; Lazar et al., 2011; Håvelsrud et al., 2012), linked to the presence of fluid and hydrocarbon seepage. In recent years, an increasing variety of hydrocarbon degrading prokaryotes has been described, mainly from the *Alpha*-, *Delta*- and *Gammaproteobacteria* class and *Firmicutes* phylum (Head et al., 2006; Kniemeyer et al., 2007; Yakimov et al., 2007; Håvelsrud et al., 2012; Jaekel et al., 2013). Members of these groups are able to degrade complex mixtures of hydrocarbons (Meckenstock and Mouttaki, 2011; Rosenberg, 2013; Abbasian et al., 2015), and often dominate communities in post-oil spill samples (Kostka et al., 2011; Mason et al., 2012; Joye et al., 2014; Kleindienst et al., 2016).

The Mediterranean Sea is a semi-closed marine system with peculiar characteristics: high deep-water temperature and homeothermy (ca. 13°C), fast deep-water turnover (in the order of 11–100 years Santinelli et al., 2010) and a strong decreasing trophic gradient moving from the Western Basin to the Eastern Basin (Koppelmann et al., 2004). Pockmarks are common features on many continental margins of the Mediterranean Basin (Hovland and Curzi, 1989; Dimitrov and Woodside, 2003; Trincardi et al., 2004; Geletti et al., 2008; Taviani et al., 2013; Taviani, 2014). Despite this, the effects of the shallow-water pockmarks of the Mediterranean basin and associated seeping on the surface sediment microbial community, remain largely unknown.

Here, we have examined the diversity and distribution of benthic microbial communities associated with shallow-water pockmarks adjacent to the Middle Adriatic Ridge. The microbial diversity data were integrated with measurements of sedimentary hydrocarbons and organic matter content. Specifically, we investigated whether: (i) shallow-water pockmarks of the Middle Adriatic shelf are associated with a sedimentary hydrocarbon anomaly; (ii) the structure of the prokaryotic community differs among pockmarks and surrounding sediments and such change is significantly correlated with sediment chemistry; and (iii) the different microbial assemblages of shallow-water pockmarks are dominated by few or several phylotypes, implying a specialized or a generalized community, respectively.

## Materials and methods

### Sampling site and procedure

**Figure 1:**
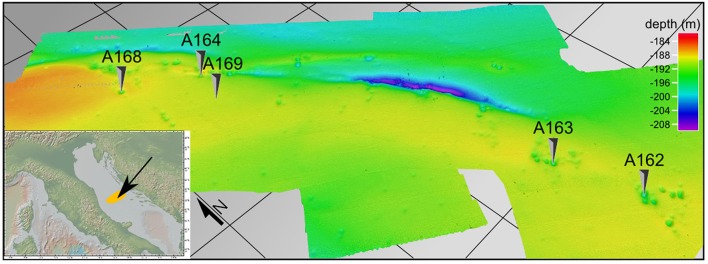
**High-resolution shaded relief DTM of the sampled pockmark field in the Middle Adriatic shelf**. Several pockmarks are visible, together with the depression associated with the MAR. Sampled pockmarks and sediments are marked with a black arrow.

Sediment samples were collected ca. 75 nautical miles South-East of Ancona (Italy) during the ARCADIA oceanographic cruise (SeARch of CorAl banks in the miDdle AdrIAtic) on board the R/V *Urania* in March 2010. The surveyed area covers ca. 27 km^2^ and harbors over 200 pockmarks in a flat area at a depth of 190 m (Figure 1), described previously by Mazzotti et al. (1987), Taviani (2014). The sampling strategy included five different stations in close proximity of the Middle Adriatic Ridge (42.8°N, 15.08°E). Stations A162, A163, A164, and A168 were situated at the center of randomly selected pockmarks, while station A169 was situated in open sediments in proximity to pockmark A164 (Figure 1 and Table 1). Three undisturbed sediment cores were collected at each station and were divided in three distinct horizons shipboard (0–1, 3–5, 10–15 cm). Surface sediment aliquots were immediately placed in gas tight vials for the determination of volatile aliphatic hydrocarbons (C5-C10). Aliquots maintained at −20°C were used for the analyses of the organic matter composition, the content of semi-volatile or non-volatile aliphatic hydrocarbons (C10-C40), monoaromatic hydrocarbons (benzene, toluene, ethyl-benzene, xylene, BTEX), polycyclic aromatic hydrocarbons (PAHs), and to extract the genomic DNA of the microbial community. Samples for the determination of prokaryotic abundance and biomass were preserved in 2% (final concentration) sterile formaldehyde. Sediment sub-samples for fluorescence *in-situ* hybridization (FISH) were fixed in 2% formaldehyde for 1 h at room temperature, washed three times with phosphate-buffered saline (PBS) solution and then stored in PBS/ethanol (1:1; v/v) at −20°C until further processing.

**Table 1:** **Details of the sampled stations and sedimentary organic matter content**.

**Station**	**Lat (°N)**	**Long (°E)**	**Depth w. col. (m)**	**Layer (cm)**	**Protein (mg g^−1^)**	**Carbohydrate (mg g^−1^)**	**Lipid (mg g^−1^)**	**BPC (mgC g^−1^)**	**CPE (μg g^−1^)**
A162	42.866	15.095	189	0	1.34 ± 0.1	0.55 ± 0.07	0.53 ± 0.03	1.27 ± 0.02	6.94 ± 0.36
				5	1.44 ± 0.1	0.44 ± 0.00	0.14 ± 0.03	0.98 ± 0.10	5.26 ± 0.20
				10	1.74 ± 0.0	0.52 ± 0.18	0.38 ± 0.06	1.35 ± 0.15	4.76 ± 0.17
A163	42.874	15.092	194	0	1.68 ± 0.2	0.37 ± 0.07	0.23 ± 0.06	1.14 ± 0.11	5.45 ± 0.30
				5	1.65 ± 0.5	0.64 ± 0.17	0.08 ± 0.02	1.12 ± 0.26	4.33 ± 0.28
				10	1.84 ± 0.2	0.49 ± 0.08	0.17 ± 0.04	1.23 ± 0.06	4.23 ± 0.34
A164	42.907	15.072	190	0	1.40 ± 0.1	0.53 ± 0.17	0.24 ± 0.06	1.08 ± 0.08	7.30 ± 0.53
				5	1.17 ± 0.2	0.45 ± 0.01	0.32 ± 0.05	0.99 ± 0.10	4.39 ± 0.20
				10	1.36 ± 0.2	0.39 ± 0.09	0.08 ± 0.00	0.89 ± 0.09	1.13 ± 0.07
A168	42.911	15.062	197	0	0.60 ± 0.1	0.49 ± 0.01	0.19 ± 0.00	0.63 ± 0.05	2.86 ± 0.21
				5	0.60 ± 0.1	1.73 ± 0.44	0.19 ± 0.04	1.13 ± 0.14	2.15 ± 0.09
				10	0.73 ± 0.1	0.33 ± 0.10	0.14 ± 0.01	0.60 ± 0.06	0.62 ± 0.11
A169	42.903	15.069	190	0	0.91 ± 0.2	0.90 ± 0.25	0.20 ± 0.00	0.95 ± 0.18	4.40 ± 0.20
				5	0.71 ± 0.2	0.50 ± 0.24	0.25 ± 0.05	0.74 ± 0.17	1.67 ± 0.34
				10	0.75 ± 0.1	0.54 ± 0.02	0.10 ± 0.01	0.66 ± 0.06	0.60 ± 0.18

Results are reported as average ± standard deviation. BPC, Biopolymeric organic carbon; CPE, Chloroplastic pigment equivalents.

### Characterization of sediment hydrocarbons

Volatile aliphatic hydrocarbons (C5-C10), semi-volatile and non-volatile aliphatic hydrocarbons (C10-C40), monoaromatic hydrocarbons (BTEX) and PAHs were analyzed in sediments by conventional procedures based on gas-chromatography with flame ionization detector (FID), gas-chromatography and mass spectrometry, and HPLC with fluorimetric detection (Piva et al., 2011; Benedetti et al., 2014; Etiope et al., 2014). Limitations in our sampling gear prevented us from analyzing volatile aliphatic hydrocarbons between C1 and C4. Briefly, for the analyses of volatile aliphatic hydrocarbons (C5-C10), samples previously stored within gas-tight sealed vials and maintained at −20°C, were warmed to 80°C for 30 min: the head space gas was analyzed by gas chromatography (Perkin Elmer Clarus 500) with an Elite-5 capillary column (30 m × 0.32 mm × 0.25 μm) and FID. Compounds were identified by standardization with pure standards of analytical GC grade *n*-pentane, *n*-hexane, *n*-heptane, and *n*-decane. Semi-volatile and non-volatile aliphatic hydrocarbons (C10-C40) were extracted from sediments with hexane:acetone (2:1) in a microwave (110°C for 25 min, 800 Watt) (Mars CEM, CEM Corporation, Matthews NC). After centrifugation at 3.000 × g for 10 min, the supernatants were purified with solid-phase extraction (Phenomenex Strata-X, 500 mg × 6 mL plus Phenomenex Strata-FL, 1000 mg × 6 mL) and then concentrated using a SpeedVac (RC1009; grade *n*-hexane and analyzed with a Perkin Elmer gas chromatograph) equipped with an Elite-5 capillary column (30 m × 0.32 mm ID × 0.25 μm-df) and a FID. For quantitative determination, the system was calibrated with an unsaturated pair *n*-alkane standard mixture according to EN ISO 9377-3 (Fluka 68281). Monoaromatic hydrocarbons BTEX, benzene, toluene, ethylbenzene, xylene (orto-, meta-, para-) were analyzed using gas chromatography and mass spectrometry. Samples were extracted with *n*-hexane:acetone (2:1) in a microwave system (Microwave Digestion and Extraction System Mars-5, CEM) at 110°C and 800 W for 25 min. After centrifugation at 3.000 × g for 10 min, the supernatants were purified by solid phase extraction (Phenomenex Strata-X, 500 mg × 6 mL plus Phenomenex Strata-FL, 1000 mg × 6 mL) and then concentrated using a SpeedVac (RC1009; Jouan, Nantes, France) to dryness. Compounds were re-dissolved in analytical GC-MS grade *n*-hexane (1 mL) and analyzed using a GC-MS system, Varian Chrompack CP-3800 equipped with Saturn 2000 ionic trap. For the analysis of PAHs, sediment samples were extracted using 0.5 M potassium hydroxide in methanol with microwave at 55°C for 20 min (800 Watt) (CEM, Mars System). After centrifugation at 3.000 × g for 10 min, the methanol extracts were concentrated using a SpeedVac and purified with solid-phase extraction (Octadecyl C18, 500 mg × 6 mL, Bakerbond). A final volume of 1 mL was recovered with pure, analytical HPLC gradient grade acetonitrile; HPLC analyses were carried out in a water-acetonitrile gradient by fluorimetric and diode array detection. The PAHs were identified according to the retention times of an appropriate pure standards solution (EPA 610 Polynuclear Aromatic Hydrocarbons Mix), and classified as low molecular weight (LMW: naphthalene, acenaphthylene, 1-methyl naphthalene, 2-methyl naphthalene, acenaphthene, fluorene, phenanthrene, anthracene) or high molecular weight (HMW: fluoranthene, pyrene, benzo(a)antrhacene, chrysene, 7,12-dimethyl-benzo(a)anthracene, benzo(b)fluoranthene, benzo(k)fluoranthene, benzo(a)pyrene, dibenzo(a,h)anthracene, benzo(g,h,i)perylene, indeno(1,2,3-cd)pyrene). Accuracy and precision were checked analyzing both pure standard solutions and reference materials (NIST 1944) and the obtained concentrations were always within the 95% confidence intervals of certified values. Aliquots of all the samples were dried in a oven at 60°C for at least 8 h, up to obtain a constant weight, in order to quantify the interstitial water content, allowing to express all the analyzed chemicals as a function of the dry weight (d.w.) of the sediments.

### Sedimentary geochemistry

Frozen samples were thawed at 25°C and porewater was separated by centrifugation (3200 rpm) and filtering of the supernatant (0.2 μm). A subsample from each depth interval was analyzed with a four-channel autoanalyzer (Seal Analytical) for nitrate+nitrite, ammonium, phosphate, and silicate using the standard colorimetric methods for nutrients (Tuğrul et al., 2014). Another subsample was used for ion chromatographic analysis. Concentrations of anions (i.e., sulfate) were determined using a Dionex AS4A-SC separation column, sodium hydroxide eluent, and ASRS-I suppressor whereas cations (i.e., magnesium) were measured with a Dionex CS12-SC separation column, methane sulfonic acid eluent and CSRS-I suppressor (Koçak et al., 2012).

### Sedimentary organic matter

Total protein, carbohydrate, lipid, chlorophyll-a, and phaeopigments were determined as previously described (Danovaro, 2010). Concentrations were calculated using standard curves, and normalized to sediment dry weight after desiccation (60°C, 24 h). Protein, carbohydrate, and lipid concentrations were converted into carbon (C) equivalents using the conversion factors of 0.49, 0.40, and 0.75 μgC μg^−1^, respectively (Fabiano et al., 1995). Biopolymeric organic carbon (BPC) was calculated as the sum of the carbon equivalents of protein, carbohydrate, and lipid, and was used as a proxy for the available trophic resources (Pusceddu et al., 2009). Chloroplastic pigment equivalents are defined here as the sum of the chlorophyll-a and phaeopigment concentrations.

### Total prokaryotic number and biomass

Total prokaryotic counts were performed using an acridine orange staining technique (Luna et al., 2002). Briefly, tetrasodium pyrophosphate was added to 0.5 g of the fixed sub-samples, which were incubated for 15 min in the dark before sonication. The samples were then stained with acridine orange (final concentration, 0.025%), filtered on 0.2 μm pore-size polycarbonate filters under low vacuum, and analyzed as described by Fry (1990) using epifluorescence microscopy (1000 × magnification). Prokaryotic counts and cell dimensions were obtained using ImageJ (Abramoff et al., 2004). In order to take into account the possible effect of cell size on the carbon content three different estimate of carbon content per unit volume were used. Cells biovolumes were converted to biomass assuming an average carbon content of 400 fg μgC μm^−3^ (Simon and Azam, 1989), 310 fgC μm^−3^ (Fry, 1990), and 133 fgC μm^−3^ (Simon and Azam, 1989). Total prokaryotic counts and prokaryotic biomass were normalized to sediment dry weight after desiccation (24 h at 60°C).

### Fluorescent *in-situ* hybridization

To investigate the abundance of *Bacteria* and *Archaea* relative to total prokaryotes, FISH was used as described by Pernthaler et al. (2002). The Cy3 oligonucleotide probes used were EUB338-mix (EUB338, 5′-GCT GCC TCC CGT AGG AGT-3′, EUB338-II, 5′-GCA GCC ACC CGT AGG TGT-3′, and EUB338-III, 5′-GCT GCC ACC CGT AGG TGT-3′), which targeted total Bacteria (Amann et al., 1990; Daims et al., 1999), ARCH915 (5′-GTG CTC CCC CGC CAA TTC CT-3′), which targeted total Archaea (Stahl and Amann, 1991) and NON338 (5′-ACT CCT ACG GGA GGC AGC-3′) as the negative control (Amann, 1995). Briefly, *Bacteria* cell-wall was permeabilized by incubating the filters in lysozyme (10 mg mL^−1^ in 0.05 M EDTA, pH 8.0; 0.1 M Tris-HCl, pH 7.5) for 60 min at 37°C, followed by three washes in MilliQ water and 1 min incubation in 96% ethanol at room temperature. *Archaea* cell-wall was permeabilized by incubating the filters in Proteinase K (0.15 μg mL^−1^ for 2.5 U mg^−1^ in 0.05 M EDTA, pH 8.0; 0.1 M Tris-HCl, pH 7.5, (Teira et al., 2004) for 60 min at 37°C, followed by three washes in MilliQ water and 1 min incubation in 96% ethanol at room temperature. Fifty ng μL^−1^ of each probe were hybridized to their targets by incubating the filter at 46°C for 2 h in hybridization buffer (0.9 M NaCl, 20 mM Tris-HCl pH 7.4, 0.01% SDS, 35% Formamide). After the hybridization the filter are washed, counter stained with DAPI (4′,6-diamidin-2-phenilindole), air dried, mounted on microscope slides and analyzed under the epifluorescence microscopy (1000 × magnification) with an appropriate set of filters. The *Bacteria* to *Archaea* ratio (BAR) was calculated as BAR = [(B−A)/(B+A)] (Giovannelli et al., 2013b), where B and A are the abundance of *Bacteria* and *Archaea*, respectively. The BAR ratio is constrained between 1 (community composed by only *Bacteria)* and −1 (community composed exclusively by *Archaea)* and is equal to 0 if the contribution of both domain is equal to 50%.

### Extraction of genomic DNA from the microbial community

Genomic DNA was extracted from frozen surface sediment aliquots of each replicated core following a modified phenol:chloroform extraction procedure (Giovannelli et al., 2013a), and replicates were pooled together before amplification to reduce potential variability. Briefly, ca. 0.5 g of sediments were added to 850 μl extraction buffer (50 mM Tris-HCl, 20 mM EDTA, 100 mM NaCl; pH 8.0) and 5 μL proteinase K (20 mg mL^−1^). Following incubation at 37°C for 30 min, 50 μl SDS (20%) were added and incubated 1 h at 65°C with occasional mixing. After centrifugation, the pellet was washed with a second aliquot of extraction buffer and the supernatants were combined. DNA was extracted by a series of phenol:chloroform:isoamyl alcohol (25:24:1) and chloroform:isoamyl alcohol (24:1) extraction. The final supernatant was precipitated in 3 M sodium-acetate and isopropanol, washed twice with 70% ice cold ethanol and resuspended in ultra-pure water. The integrity of 16S rRNA genes was assessed by polymerase chain reaction amplification using the bacterial primers 27f-1517r (Vetriani et al., 2004). The product was visualized on 1% agarose gel stained with ethidium bromide. Multiple extractions were combined to reduce potential bias.

### 16S rRNA gene amplification and tag-encoded FLX amplicon pyrosequencing

The diversity of the prokaryotic community was assessed using the variable 4 (V4) region of the 16S rRNA gene targeted with prokaryotic universal primers (515f 5′-GTG CCA GCM GCC GCG GTA A-3′ and 806r 5′-GGA CTA CVS GGG TAT CTA AT-3′, Caporaso et al., 2011) and subjected to amplicon pyrosequencing at the Molecular Research LP facilities (Shallowater, TX, USA). Multiple PCR reactions were combined to reduce potential bias. Sequences are available through the NCBI Short Read Archive database with accession number SRP051591.

### Statistical and bioinformatic analyses

Statistical analyses were performed with the statistical R-software (R Development Core Team, 2010). The samples were investigated for differences in measured variables among the sampled stations and sediment layers using ANOVA. Where ANOVA assumptions were rejected, a more conservative level of p was chosen (Underwood, 1991). In case of significant differences, a HSD Tukey *post-hoc* test was performed. Generated 16S rRNA gene sequences were processed and analyzed using the QIIME 1.7 software package (Caporaso et al., 2010b). Briefly, sequences shorter than 200 bp, containing unresolved nucleotides, exhibiting an average quality score lower than 25, harboring mismatches longer than 2 bp in the forward primer, or possessing homopolymers longer than 8 bp were removed with the QIIME script split_libraries.py. This script also removes forward and reverse primer sequences and splits samples according to the tag. Subsequently, pyrosequencing noise was removed by employing the Denoiser 0.91 (Reeder and Knight) included in QIIME. Chimeric sequences were removed using UCHIME (Edgar et al., 2011), included in USEARCH (6.0.152). As the studied environment might harbor *Bacteria* and *Archaea*, which are not well represented by reference databases, we applied UCHIME in *de novo* mode. Subsequently, we used UCHIME in reference mode against the Greengenes Gold dataset (gold_strains_gg16S_aligned_19-Mar-2011.fasta) as the reference database (DeSantis et al., 2006; Edgar et al., 2011). Operational taxonomic unit (OTUs) determination was performed with the UCLUST algorithm (Edgar, 2010) at 97% similarity (Schloss and Handelsman, 2005) and a representative set selected employing the QIIME scripts pick_otus.py and pick_rep_set.py (Caporaso et al., 2010a). Taxonomic classification of selected reference sequences (OTUs) was performed by similarity searches using the Ribosomal Database Project classifier (Wang et al., 2007) included in QIIME. Diversity estimates (Chao1) were calculated based on rarefaction of 3000 randomly chosen sequences. Shared and unique OTUs were identified using the core_microbiome.py and make_otu_network.py QIIME script. The results were parsed with custom scripts, and used together to visualize and analyze the community network with Gephi (Bastian et al., 2009) and the Yifan Hu layout, a multilevel, force directed algorithm (Hu, 2005). The network structure and OTU table were employed in combination with Circos (Krzywinski et al., 2009) to visualize community composition, unique and shared OTUs and depth of taxonomic assignment. The phylogenetic tree obtained with PyNAST (Caporaso et al., 2010a) was used in Topiary Explorer (Pirrung et al., 2011) to visualize the community with tree branches collapsed at 80% of taxonomic consensus. The OTU table was imported in R to perform non-metric multi-dimensional scaling (nMDS) and cluster analysis using the Jaccard dissimilarity and the average linkage method in Vegan (Oksanen et al., 2012). Publicly available 16S rRNA pyrotag datasets that were sequenced with the same set of primer were used for comparative purposes, and reanalyzed together with our libraries in QIIME using the same procedure. The following datasets were combined: SRS290213 (Patagonia coastal sediments), SRR192300 (Rainbow-6 deep-sea hydrothermal vent, Flores et al., 2011), DRR001439, and DRR001438 (deep-sea methane seeps at the Nankai Trough, Nunoura et al., 2012).

## Results

### Geochemistry, hydrocarbon, and organic matter composition of pockmark sediments

The sampled stations in this study are in proximity of a pinch-like depression oriented NW-SE and situated on the Mid-Adriatic Ridge (Finetti et al., 1987; Mazzotti et al., 1987). All sampled stations follow this NW-SE axis (Figure 1 and Table 1). Pockmark A164 is aligned with the pinch-like depression, while all other pockmark stations are situated slightly off axis. Pockmarks range from 50 to 200 m in diameter and 1 to 6.5 m in depth; they are randomly distributed in muddy sediments of the surveyed area, and are at times clustered in groups of 5–7. The shape is circular and annular and sometimes they are coalescent.

**Figure 2:**
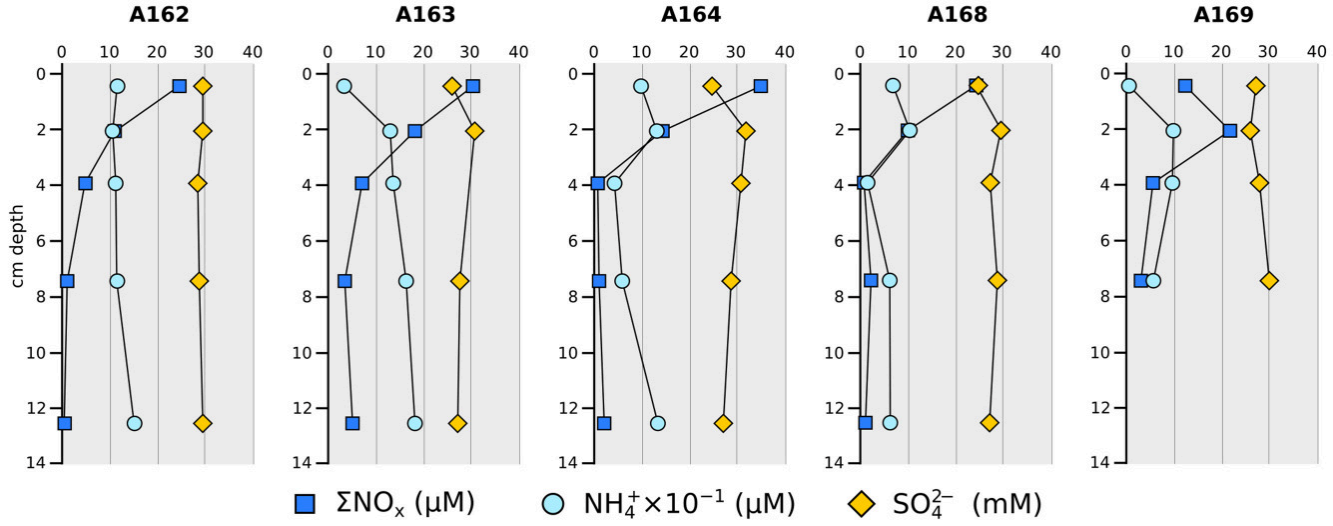
**Vertical profiles of selected chemical constituents in sediment porewaters**. ∑NO_*x*_ = ${\text{NO}}_{3}^{-}$ + ${\text{NO}}_{2}^{-}$.

Sedimentary concentrations of nutrients and major cations measured in the sediment porewaters are reported in Table 2 and Figure 2. All examined cores displayed a downcore depletion of nitrate and nitrite (∑NO_x_; Figure 2). Nitrate concentrations in the cores from station A164 and A168 reached close to zero already at the 3–5 cm layer, while the other stations had the nitrate minimum in deeper layers. Station A169 had lower nitrate concentrations in the surface sediments then the other sampled stations (Figure 2). All sampled station showed a downcore accumulation of ammonium while sulfate levels were more or less constant, except for the very top layer in A163, A164, and A168. Silica and phosphate concentrations also increased with increasing sediment depth in all investigated stations (Table 2). Concentrations of all other major cations did not show strong trends downcore, and were not different between stations. Pockmark station A164 was the only exception with a downcore increase in the concentrations of potassium, magnesium, and calcium.

**Table 2:** **Summary of the geochemical species measured in the sediment pore water along the sedimentary profile**.

**Station**	**Layer (cm)**	**∑NO_x_ (μM)**	**${\text{NH}}_{4}^{+}$ (μM)**	**${\text{SO}}_{4}^{2-}$ (mM)**	**${\text{PO}}_{4}^{3-}$ (μM)**	**Si (μM)**	**Br (μM)**	**Na^+^ (mM)**	**K^+^ (mM)**	**Mg^2+^ (mM)**	**Ca^2+^ (mM)**
A162	0–1	23.5	119.2	29.6	2.0	70.7	707.4	533.3	9.3	51.3	8.7
	1–3	11.1	109.0	29.8	2.2	95.8	717.3	532.4	9.7	62.6	9.4
	3–5	4.6	116.2	28.6	2.3	120.5	687.9	519.2	7.6	59.7	9.1
	5–10	1.2	116.3	28.9	2.8	177.5	703.1	525.1	10.3	63.6	13.8
	10–15	0.6	146.1	29.6	3.1	168.8	711.3	522.9	9.9	62.7	9.2
A163	0–1	30.6	35.7	26.4	1.9	87.1	637.2	483.2	8.5	45.2	9.3
	1–3	18.3	117.2	29.5	2.4	100.0	703.8	521.1	8.5	60.5	8.6
	3–5	8.8	129.7	Na	2.6	144.1	na	na	na	na	na
	5–10	4.2	164.9	28.6	2.5	147.5	682.3	512.8	9.7	58.5	9.8
	10–15	4.5	180.5	28.2	3.3	182.6	684.6	520.4	8.7	58.9	11.2
A164	0–1	33.4	99.0	23.8	2.2	73.9	589.2	448.2	8.0	37.5	6.6
	1–3	13.8	121.7	31.2	2.6	98.8	752.5	549.6	9.6	61.8	9.4
	3–5	0.6	45.0	31.1	2.7	119.2	751.6	555.5	9.7	61.8	9.1
	5–10	0.6	63.0	30.5	3.0	131.4	734.8	540.2	9.1	60.7	10.4
	10–15	1.9	127.8	29.4	3.5	172.7	715.7	543.2	9.3	62.0	9.5
A168	0–1	24.6	74.3	25.9	2.1	57.9	628.6	462.7	8.2	41.3	8.4
	1–3	9.3	102.1	29.9	2.4	100.6	724.1	538.1	8.8	65.9	9.4
	3–5	0.6	3.6	28.6	2.9	125.6	694.8	515.9	9.0	64.0	9.0
	5–10	0.9	43.4	29.7	3.0	126.4	724.1	536.4	9.6	69.7	9.9
	10–15	0.8	45.2	27.3	3.2	123.6	672.0	505.2	8.5	60.2	8.7
A169	0–1	11.3	1.5	28.5	2.4	73.2	690.2	505.0	9.4	44.2	8.4
	1–3	21.3	98.4	27.8	2.5	68.8	889.0	668.7	10.2	68.6	11.2
	3–5	5.4	97.5	29.3	2.9	96.3	709.1	518.0	9.0	53.8	9.8
	5–10	2.0	66.4	30.4	3.2	114.1	749.1	556.2	9.3	60.5	9.2
	10–15	na	na	Na	na	na	na	na	na	na	na

∑NO_x_ = ${\text{NO}}_{3}^{-}$ + ${\text{NO}}_{2}^{-}$; na = not available.

Sedimentary concentrations of volatile aliphatic hydrocarbons (C5-C10), semi-volatile and non-volatile aliphatic hydrocarbons (C10-C40), monoaromatic hydrocarbons (BTEX), and PAHs were measured in the pockmarks and surrounding sediments to investigate the extent and nature of seepage activity (Table 3). Differences among sampled stations were observed for volatile aliphatic hydrocarbons (C5-C10), with pockmark A164 having concentrations ca. two-fold higher compared to the other stations (Table 4, ANOVA *p* < 0.001). Semi-volatile and non-volatile aliphatic hydrocarbons (C10-C40) showed comparable values and no significant differences among the stations. Monoaromatic hydrocarbon (BTEX) concentrations were higher in stations A164 and A169; these results were mostly due to the elevated content of toluene, ethyl-benzene and xylene, which showed similar concentrations in the deeper layers of such pockmarks, while benzene always remained below detection limits. PAHs were dominated by the low molecular weight fraction (from naphthalene to anthracene) with concentrations in the order of 90–110 ng g^−1^ (d.w.) of sediment, and no relevant differences between sampling sites.

**Table 3:** **Summary of hydrocarbon concentrations in sampled stations integrated over the sediment layer: C5-C10, volatile aliphatic hydrocarbons from C5 to C10; C10-C40, semi-volatile and non-volatile aliphatic hydrocarbons from C10 to C40; BTEX, monoaromatic hydrocarbons (benzene, toluene, ethyl-benzene, xylene); PAH, polycyclic aromatic hydrocarbons, low molecular weight (low), high molecular weight (high), and total (tot)**.

**Station**	**C5-C10 (ng g^−1^)**	**C10-C40 (μg g^−1^)**	**BTEX (ng g^−1^)**	**PAH _low_ (ng g^−1^)**	**PAH_high_ (ng g^−1^)**	**PAH_tot_ (ng g^−1^)**
A162	158.5 ± 31	29.2 ± 4.9	31.6 ± 7.6	98.5 ± 41.2	10.1 ± 2.8	108.6 ± 43.9
A163	192.2 ± 153.9	25.4 ± 11.8	15.6 ± 12.1	94 ± 17	4.7 ± 0.8	98.6 ± 16.2
A164	438.6 ± 169.3	29.5 ± 9.6	181.5 ± 253.9	92.7 ± na	5.1 ± na	97.8 ± na
A168	152.9 ± 6.4	24.9 ± 8.3	<0.5	90.7 ± 21.6	15.7 ± 15.3	122.9 ± 9.1
A169	161.8 ± 5.4	22.4 ± 8.0	278.3 ± 149.4	110.9 ± 5.6	10.1 ± 6.7	121 ± 2.2

Mean value and standard deviation are reported, concentrations are referred to the dry weight (d.w.) of the sediments; na, not available.

**Table 4:** **Results of ANOVA analysis on the main measured variables**.

**Factor**	**PRT**		**BPC**		**CPE**		**TPN**		**PBM**		**BAR**		**Active**		**C5-C10**	
	***F***	***p***	***F***	***p***	***F***	***p***	***F***	***p***	***F***	***p***	***F***	***p***	***F***	***p***	***F***	***p***
Stations	49.2	^***^	23.4	^***^	335.8	^***^	17.0	^***^	17.3	^***^	8.9	^***^	6.7	^***^	50.3	^***^
Layer	2.8	ns	1.3	ns	520.9	^***^	104.2	^***^	104	^***^	10.2	^***^	5.7	^**^	NA	NA
Station^*^Layer	1.0	ns	7.6	^***^	43.7	^***^	7.5	^***^	7.5	^***^	11.2	^***^	0.9	ns	NA	NA

PRT, Proteins; BPC, Biopolymeric organic carbon; CPE, Chloroplastic pigment equivalents; TPN, Total prokaryotic number; PBM, Prokaryotic biomass; BAR, Barcteria to Archaea ratio; Active, Fraction of active Prokaryotes; C5-C10, Volatile aliphatic hydrocarbons. NA, not available; ns, p > 0.05;

* p < 0.05;

** p < 0.01;

*** p < 0.001.

**Figure 3:**
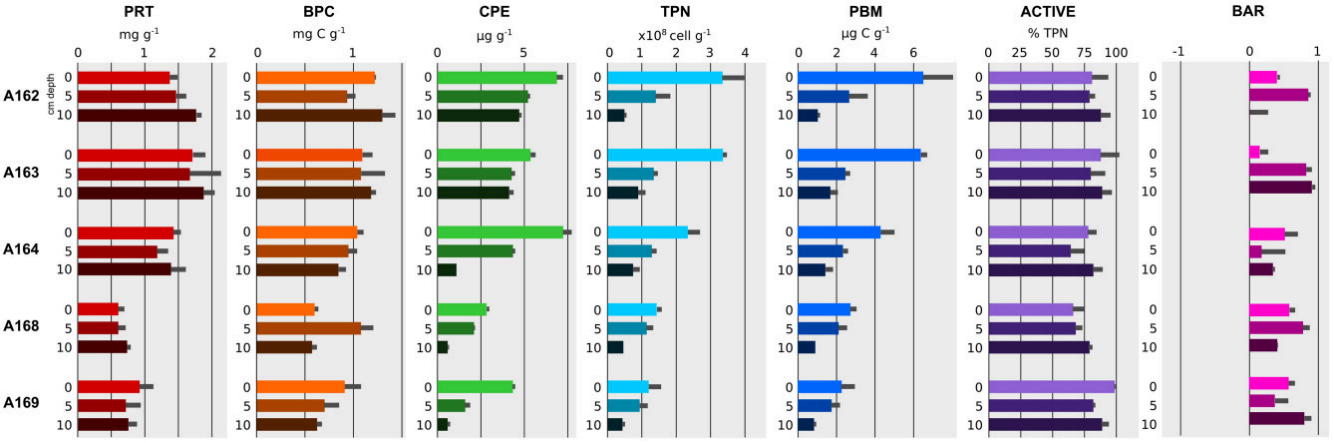
**Profile of trophic and prokaryotic variables across the sampled stations and sediment depths: PRT, Proteins; BPC, Biopolymeric organic carbon; CPE, Total phytopigments; TPN, Total prokaryotic number; PBM, Prokaryotic biomass; ACTIVE, percentage of metabolically active cells; BAR, *Bacteria* to *Archaea* ratio**. Mean value and standard deviation (dark gray bar) are reported.

Sampled stations differed in their sedimentary organic matter content (Figure 3 and Table 1). BPC concentrations ranged from 0.60 ± 0.06 to 1.27 ± 0.02 mgC g^−1^ sediment (average ± standard deviation) and were significantly higher at the pockmark stations A162, A163, and A164 (ANOVA *p* < 0.001, Table 4). Differences between sediment surface and deeper layers were found only in station A164 and A169 (ANOVA *p* < 0.001, Table 4 and Figure 3). Proteins were the dominant class of organic matter, with concentrations ranging from 0.60 ± 0.1 to 1.84 ± 0.2 mg g^−1^ sediment and followed a similar trend with higher concentrations in pockmarks A162, decreasing toward NW and the surrounding sediments (Figures 1, 2). No significant differences in protein concentrations were found between the surface sediments and the deeper layers (Table 4). Chloroplastic pigment equivalents showed a higher variability between stations and sediment layers (Figure 3 and Table 1), with higher concentration in pockmark A164 and a decreasing trend along the vertical sediment profile in all sampled stations.

### Diversity and distribution of the microbial assemblages

Sampled stations differed in terms of abundance, biomass, and BAR (Figure 3). Prokaryotic abundances (performed with Acridine orange staining) were found to be higher in the surface sediments of pockmark A162, showing a significant decreasing trend moving NW and into surrounding sediments (ANOVA, *p* < 0.001, Table 4). Prokaryotic abundances ranged from a minimum of 0.44 ± 0.08 × 10^8^ cell g^−1^ sediment in the deeper layer of station A169 to a maximum of 3.31 ± 0.8 × 10^8^ cell g^−1^ sediment in the surface sediments of pockmark A162 (Figure 3). Prokaryotic abundances decreased along the vertical profile in all stations (ANOVA, *p* < 0.001, Table 4). Prokaryotic biomass followed a similar trend, with higher values in the surface sediments of pockmark A162 and lower in the deeper layer of the surrounding sediment. The difference in calculated prokaryotic biomass values between the low and high end estimated carbon content was a factor of 3. Low end estimate of prokaryotic biomass were between 1 and 2.8 μgC g^−1^ for surface sediments, while high end estimate were as high as 8.54 μgC g^−1^ sediment. Prokaryotic biomass obtained with a unit volume carbon content of 310 fgC μm^−3^ (Fry, 1990) are reported in Figure 3.

The relative abundance of *Bacteria* and *Archaea* in the prokaryotic community was measured by mean of FISH. Active cells calculated as the contribution of total FISH targeted cells (*Bacteria* plus *Archaea*) to total prokaryotic abundance was on average of 81.6 ± 9.2%, and varied significantly among sampled stations and sediment layers (ANOVA, *p* < 0.001 and *p* < 0.01 respectively; Table 4), without showing any consistent trend (Figure 3). The BAR showed a community numerically dominated by *Bacteria* in all sampled stations and sediment layers, with the exception of the deeper layer of the pockmark station A162 (10–15 cm depth), where the community was almost evenly divided between *Bacteria* and *Archaea* (BAR 0.03 ± 0.05). BAR varied significantly among sampled stations and sediment layers (Table 4). Surface sediments of pockmark A163 showed a significantly lower BAR compared to the other surface sediments, with an average *Archaea* contribution of 38.23%.

**Figure 4:**
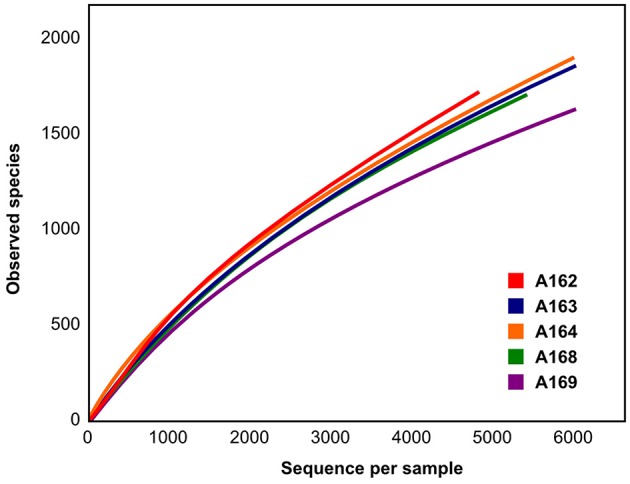
**Rarefaction curve for the 16S rRNA gene pyrotag sequences**.

**Figure 5:**
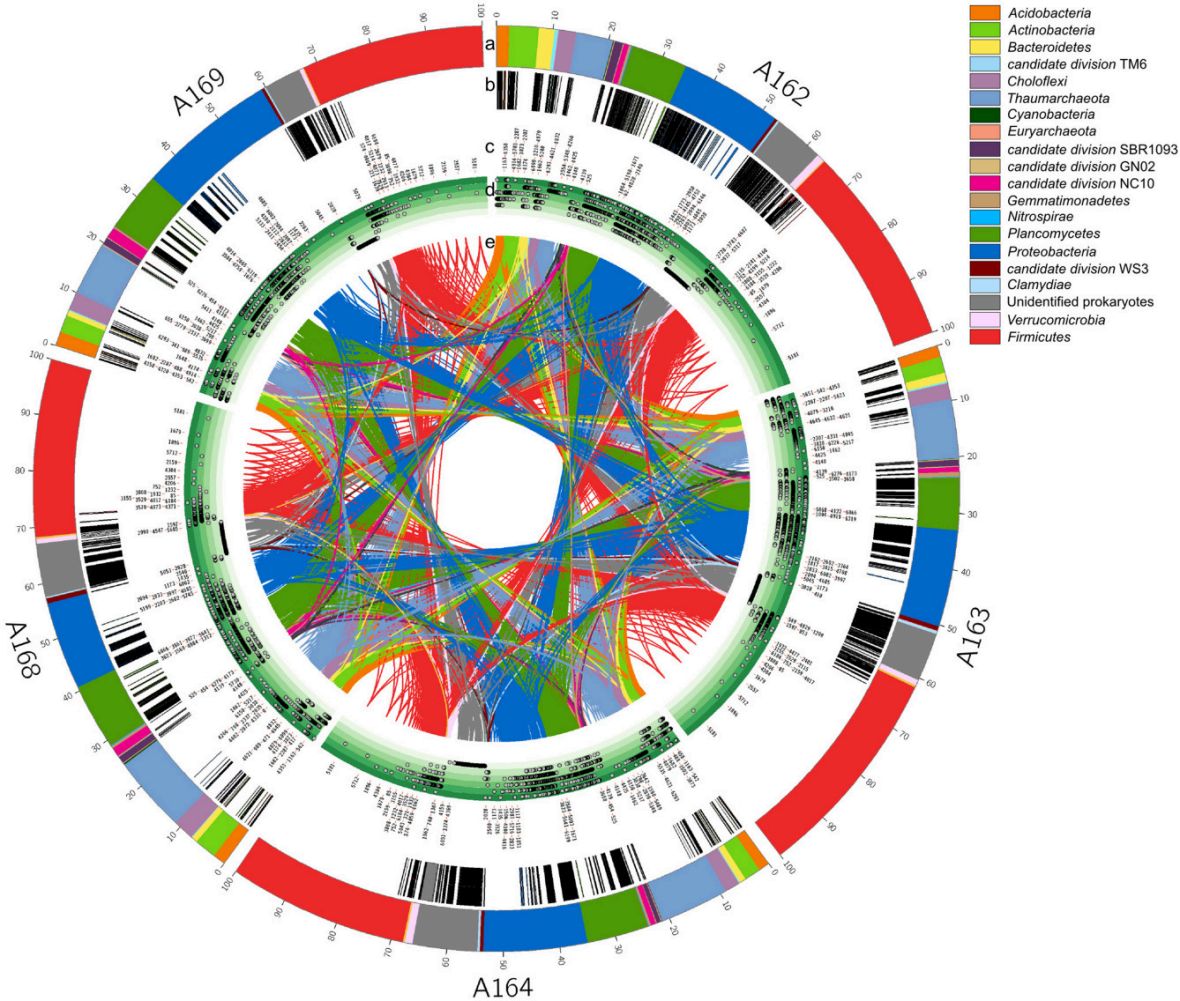
**Prokaryotic diversity in the top sediments revealed by 16S rRNA gene pyrotag sequencing analysis**. Moving from the outer circle inward: a, phylum community composition and relative abundance (%); b, location and density of library-specific OTUs (i.e., present in only one station) within each phylum; c, OTUs_ID are reported for all the abundant OTU (above 0.1% relative abundance); d, level of phylogenetic identification from kingdom to genus: the position of the dot shows the depth of phylogenetic identification for each OTUs from the lower level (inner light green circle = unclassified prokaryotes) to the higher (outer dark green circle = genus); e, connectome: network analysis highlighting the connection among OTUs shared between sampled stations. The figure was drawn using Circos (Krzywinski et al., 2009).

The prokaryotic diversity in surface sediments (0–1 cm layer) was assessed by mean of tag-encoded FLX amplicon pyrosequencing of the variable region V4 of the 16S rRNA gene with prokaryotic universal primers. A total of 30,200 sequences were generated and clustered in 5916 OTUs. Of the total obtained sequences, 9.1% could not be assigned to any known phylum, while of the remaining sequences 10.7% were classified as *Archaea* and 80.2% as *Bacteria*. The number of unclassified prokaryotic sequences was lower in station A169 than the average of pockmark stations (6.7% against 9.75%). Rarefaction curve analysis showed that, on average, pockmark stations had higher prokaryotic diversity if compared to surrounding sediments (Figure 4). Despite these differences in total diversity, the sampled stations showed a similar community structure of the most abundant phyla (Figure 5, circle a). The community was numerically dominated by the *Firmicutes* (34.1% on average), followed by the *Proteobacteria* (18.9%), *Planctomycetes* (10.5%), and *Thaumarchaeota* (10.5%). The sampled stations showed small differences in the relative abundance of these Phyla, with station A169 having the higher percentage of *Proteobacteria* (24.8%) and station A168 having the higher abundance of *Thaumarchaeota* (13.6%). Overall, 34 phyla were identified, with 14 candidate divisions with no clear phylogenetic position. All major marine phyla were represented, with 21 of those passing the 0.1% cut-off and only 11 with abundances above 1% (Table 5).

**Table 5:** **Phyla with relative abundance above 0.1% in surface sediments 16S rRNA gene libraries**.

	**Abundance**					
**Phylum**	**Average (%)**	**A162 (%)**	**A163 (%)**	**A164 (%)**	**A168 (%)**	**A169 (%)**
**ABOVE 1%**						
*Firmicutes*	34.10	35.90	38.20	32.10	31.70	32.50
*Proteobacteria*	18.90	18.20	17.30	18.50	15.70	24.80
*Thaumarchaeota*	10.50	6.60	10.60	12.40	13.60	9.50
*Planctomycetes*	10.50	10.10	9.20	10.80	11.60	10.60
Unc. Prokaryotes	9.10	9.00	8.70	11.60	9.50	6.60
*Actinobacteria*	3.60	5.20	2.90	3.00	4.00	3.10
*Chloroflexi*	3.20	3.20	2.80	2.70	3.80	3.30
*Acidobacteria*	2.20	2.30	2.00	2.00	2.20	2.60
*Bacteroidetes*	1.50	2.80	1.50	1.20	1.10	0.90
NC10	1.30	0.90	0.90	1.00	1.60	2.00
*Gemmatimonadetes*	1.20	1.50	1.00	0.80	1.30	1.20
*Verrucomicrobia*	1.00	1.40	1.00	1.30	0.80	0.70
**BETWEEN 1 AND 0.1%**						
SBR1093	0.60	0.50	0.70	0.60	0.80	0.50
*Nitrospirae*	0.50	0.40	0.90	0.40	0.40	0.30
TM6	0.40	0.30	0.60	0.40	0.30	0.30
*Chlamydiae*	0.30	0.60	0.30	0.10	0.10	0.20
WS3	0.30	0.20	0.40	0.40	0.30	0.20
*Euryarchaeota*	0.20	0.20	0.20	0.20	0.10	0.10
OP3	0.20	0.00	0.20	0.30	0.10	0.20
*Cyanobacteria*	0.10	0.10	0.10	0.10	0.20	0.00
GN02	0.10	0.10	0.20	0.10	0.10	0.10
NKB19	0.10	0.10	0.10	0.10	0.20	0.00

Within the *Firmicutes* phylum, the *Bacilli* class dominated the retrieved sequences in all samples, with an average relative contribution of 34%. The most abundant OTU (OTU 5181) within the phylum constituted on average 19.8% of the retrieved sequences, and was related to *Bacillus aquimari*s (accession number AF483625.1, 99.2% similarity, Table 6), a common Gram-positive whose closest cultured relatives have been isolated in different environmental settings. The *Proteobacteria* phylum was numerically dominated by the class *Gammaproteobacteria*, and mostly represented by the order *Alteromonadales.* Sequences related to this group significantly differed in abundance among stations reaching 7.8% in sediment station A169, while comprised between 0.001 and 1.3% (average 0.4%) in the pockmarks. These sequences were represented by a single dominant OTU (OTU 5045), related to *Alteromonas* sp. M71_D56 (acc. num. FM992724.1, 99% similarity) isolated from the Eastern Mediterranean deep-sea. Members related to the genus *Thioprofundus*, within the order *Chromatiales*, were the second most abundant group within the *Gammaproteobacteria*, and represented on average 2.3% of the retrieved sequences, with an average similarity to *Thioprofundus lithotrophicum* of 95% (acc. num. AB468957). *Alphaproteobacteria* and *Deltaproteobacteria* were the second most abundant class within the *Proteobacteria* with relative average abundances of 3.8 and 2.9%, respectively. Sequences in the *Alphaproteobacteria* class were mainly related to the *Rhizobiales* and *Rhodospirillales* order. Most of the retrieved *Deltaproteobacteria* were related to the *Mixococcales* and candidate division NB1-j, amounting collectively to 1.9% of the total sequences. Members of the *Desulfuromonadaceae, Nitrospinaceae*, and *Geobacteraceae* families were also recovered (although with relative abundances below 0.1%). Sequences related to the synthrophic sulfate reducers order *Syntrophobacterales*, amounting on average to 0.25% of the reads, were also retrieved in the libraries. *Betaproteobacteria* and *Epsilonproteobacteria* related sequences were rare in all investigated samples, with relative abundances on average of 0.14 and 0.02% respectively. *Betaproteobacteria* were absent from station A163, while *Epsilonproteobacteria* related sequences were represented by singleton in all samples with the exception of pockmark A164. We also identified in our libraries the presence of the candidate division NC10 (on average 1.3% of the community), with an average similarity of 89.5% to *cadidatus* Methylomirabilis oxifera. Sequences related to the *Thaumarcheota* phylum were the third most abundant group in all sample sediments, with higher relative abundances in pockmark station A162 and A164. The average similarity to *candidatus* Nitrosopumilus maritimus (acc. num. CP000866.1) and *candidatus* Nitrosoarchaeum limnia (acc. num. CM001159.1) was 92.2 and 91.3%, respectively.

**Table 6:** **Taxonomic assignment and closest relative of the OTUs with relative abundance above 1% in any of the sampled station**.

**#OTU ID**	**Relative Abundance^a^**					**QIIME Assigned taxonomy**			**Closest Relative**	**Identity (%)**	**Acc. Num**.
	**A162**	**A163**	**A164**	**A168**	**A169**	**Phylum**	**Class**	**Order**			
4148	1.74	2.67	2.83	2.55	1.8	*Thaumarchaeota*	–	*Cenarchaeales*	*Ca*. Nitrosopumilus koreensis	97	NR_102904.1
4425	0.44	0.82	0.94	1.80	0.79	*Thaumarchaeota*	–	*Cenarchaeales*	*Ca.* Nitrosopelagicus brevis	97	CP007026.1
5181	12.32	10.92	10.26	7.12	5.79	*Firmicutes*	*Bacilli*	*Bacillales*	*Bacillus aquimaris*	99	AF483624.1
1896	3.73	3.81	3.24	2.85	3.10	*Firmicutes*	*Bacilli*	*Bacillales*	*Fictibacillus arsenicus*	100	KP307782.1
5712	4.38	3.74	3.64	2.46	2.31	*Firmicutes*	*Bacilli*	*Bacillales*	*Bacillus vietnamensis*	98	KP713658.1
2557	1.72	3.67	0.40	1.68	3.18	*Firmicutes*	*Bacilli*	*Bacillales*	*Bacillus baekryungensis*	98	KR045741.1
1679	0.91	2.62	1.52	2.90	1.46	*Firmicutes*	*Bacilli*	*Bacillales*	*Bacillus oceanisediminis*	99	LN774318.1
4304	1.87	2.05	1.99	1.94	1.43	*Firmicutes*	*Bacilli*	*Bacillales*	*Bacillus idriensis*	99	KR922323.1
2159	0.08	0.40	0.81	2.17	3.17	*Firmicutes*	*Bacilli*	*Bacillales*	*Bacillus decolorationis*	99	KF815234.1
4206	0.60	1.00	0.08	1.11	1.33	*Firmicutes*	*Bacilli*	*Bacillales*	*Sporosarcina soli*	99	AB682453.1
5045	n.d.	0.93	n.d.	n.d.	6.83	*Proteobacteria*	*Gammaproteobacteria*	*Alteromonadales*	*Salinimonas lutimaris*	99	NR_109101.1
1173	0.83	0.97	1.07	0.69	0.71	*Proteobacteria*	*Gammaproteobacteria*	*Chromatiales*	*Thioprofundum lithotrophicum*	96	NR_112829.1
2203	0.21	0.21	n.d.	0.11	2.76	*Proteobacteria*	*Gammaproteobacteria*	*Oceanospirillales*	*Microbulbifer okinawensis*	99	JQ765866.1
1435	0.02	0.07	0.65	0.81	1.71	*Proteobacteria*	*Gammaproteobacteria*	*Pseudomonadales*	*Psychrobacter maritimus*	99	KR709318.1
2540	n.d.	0.04	1.25	1.79	0.08	*Proteobacteria*	*Gammaproteobacteria*	*Pseudomonadales*	*Psychrobacter piscatorii*	99	KP715892.1
4139	1.02	0.44	0.47	0.76	0.75	*Gemmatimonadetes*	*Gemmatimonadetes*	−	*Ectothiorhodospira salini*	86	NR_104503.1
4159	n.d.	n.d.	2.33	n.d.	n.d.	Unclassified Prokaryotes	−	−	*Ammoniphilus oxalaticus*	83	Y14579.1

n.d., not detected.

a Relative abundance expressed as percentage of the total sequences for each station.

**Figure 6:**
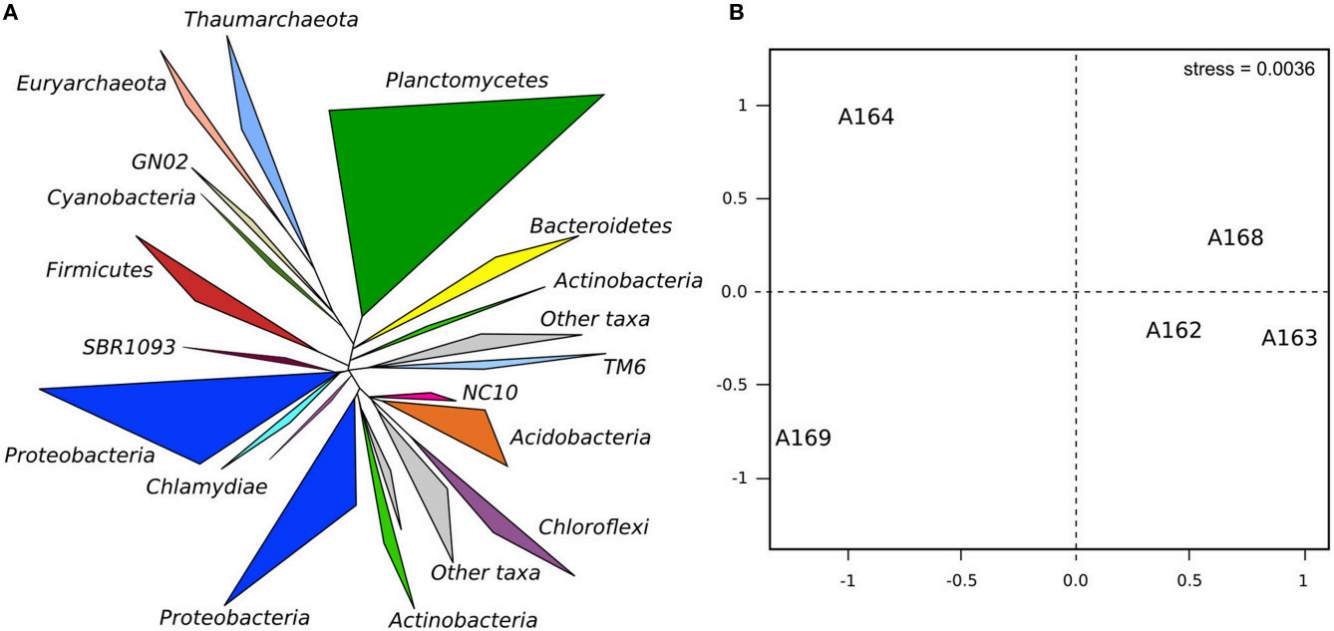
**Phylogenetic diversity within the sampled stations and the results of nMDS analysis on the diversity: (A) phylogenetic tree showing the diversity retrieved during the study**. The branches were collapsed at 80% consensus lineage, and the thickness of the branch is proportional to the number of OTUs represented; **(B)** results of the nMDS analysis based on 16S rRNA gene frequencies in the samples highlighting the differences among sampled pockmarks and flanking sediments.

**Figure 7:**
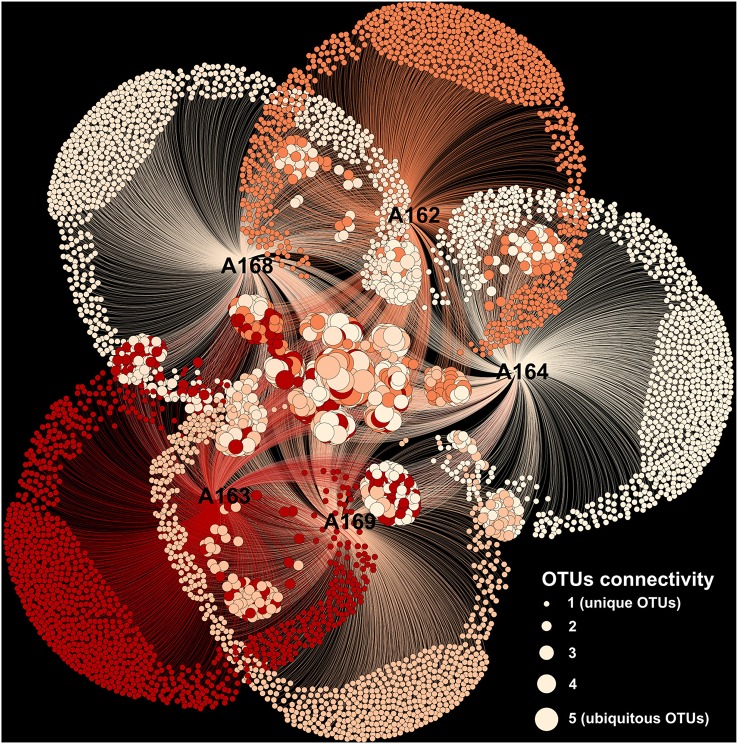
**Network analysis of the OTUs composition for each sampled station**. The network was visualized using the Yifan Hu algorithm. OTUs are color-coded based on the station. The dimension of the circle is proportional to the in-degree connection, i.e., the number of station in which the OTU was detected.

The relative abundance of rare phyla differed significantly among station. Cluster and nMDS analyses carried out on the microbial diversity revealed that sediment station A169 clustered on its own, as did pockmark station A164, while pockmark station A162A, A163, and A168 shared a more similar community (Figure 6B). Network analysis revealed that the majority of identified OTUs were station specific (Figure 7). The number of specific OTUs (i.e., present only in one of the libraries) was equal to 4405 (of 5916 OTUs total). Station A162, A163, and A164 had the highest number of specific OTUs and were not evenly distributed among taxa.

## Discussion

Hydrocarbon fluid venting in the Adriatic Sea (Mediterranean Sea) is a well-known phenomenon (Hovland and Curzi, 1989; Conti et al., 2002; Curzi, 2013, and references therein), as gas reservoirs are widely distributed in the continental shelf in the Plio-Pleistocene sediments (Stefanon, 1967; Curzi and Veggiani, 1985; Mazzotti et al., 1987). According to the literature the presence of “bubbling” and “smoke” on the surface was previously reported for the study area by fishermen in the 1970's (Conti et al., 2002; Curzi, 2013) indicating active seeping. Despite several studies describing the pockmarks features and the underlying geological structures of this area (Stefanon et al., 1983; Curzi and Veggiani, 1985; Mazzotti et al., 1987; Hovland and Curzi, 1989; Trincardi et al., 2004; Geletti et al., 2008), there is little information available on the nature of seepage, or the impact on the microbial community. We present here a multidisciplinary investigation of the pockmark field in proximity of the Middle Adriatic Ridge, integrating analyses on the sedimentary geochemistry and hydrocarbon and organic matter composition with the investigation of the diversity and distribution of the microbial assemblages.

Previous studies linked pockmarks in the Central Adriatic to gas escape in connection with halokinetic activity of deep-seated Triassic evaporites, as evidenced by the presence of bright spots and gas chimney in the sediment underling the pockmarks above and around the salt structure that forms the Middle Adriatic Ridge (Geletti et al., 2008). The stations sampled in this study were part of coalescent pockmarks and linear pockmark clusters (Figure 1), with the exception of station A168 (a unit pockmark) and station A169 situated in the surrounding sediments. The presence of coalesced pockmarks suggests the presence of periodic, rather than continuous, gas or fluid bursts through the seafloor (Pilcher and Argent, 2007; Hovland et al., 2010). Similar episodic events have been previously described for cold-seeps and mud volcanoes (Newton et al., 1980; Pilcher and Argent, 2007; Hovland et al., 2010), and the temporal variability in fluid emissions may greatly influence sedimentary chemistry and the diversity and distribution of microbial communities.

The sampled stations did not differ greatly in the downcore profile of nutrients and major cations (Figure 2 and Table 2). All stations present a rapid depletion of nitrate+nitrite moving downcore, consistent with the reduction of reactive oxidized nitrogen coupled to organic matter degradation (Devol, 2015) or potentially nitrate-driven anaerobic oxidation of hydrocarbons (see below). In station A164 and A168 the concentrations of nitrate+nitrite quickly dropped below 1 μM in the 3–5 cm sediment layer, suggesting a faster use of the oxidized nitrogen species in these stations. Overall downcore depletion of nitrate+nitrite is consistent with denitrification pathways, which probably dominated on the top 4–5 cm of the sediment. This could also explain the downcore accumulation of ammonium indicating that release from organic matter degradation was dominant over other ammonia-consuming processes. Supporting this idea is the downcore steady increase in phosphate and silica, which are also released from organic matter degradation (Dale et al., 2014). The lower ammonia concentration on the surface sediments of stations A163 and A169 could be linked to bacterial consumption, precipitation, and/or an increased rate of diffusion near the surface.

Sulfate levels were more or less constant between stations and moving downcore, with the exception of the surface sediments of stations A163, A164, and A168 (Figure 2). In these stations there was a lower surface sulfate concentration compared to stations A162 and a169. Also a slight decrease in sulfate concentrations moving downcore was present, which could indicate sulfate reduction processes.

Hydrocarbon concentrations in the sampled sediments were on average 1 order of magnitude lower compared to the few records of sedimentary hydrocarbons measured in deep-sea pockmarks worldwide (Olu-Le Roy et al., 2004; Boitsov et al., 2009; Nickel et al., 2013), but generally higher than those typical of pristine coastal areas (Benedetti et al., 2014; Etiope et al., 2014). In particular, concentrations of volatile hydrocarbons (C5-C10) and BTEX detected in A164 and A169 sediments (Table 3) were significantly higher also if compared to coastal areas with documented seepage activities of gas and oil (Benedetti et al., 2014; Etiope et al., 2014). The highest levels of volatile hydrocarbons found in pockmark A164 (2.5-fold higher than other stations) could be explained by the position of this site, situated directly above the Middle Adriatic Ridge, where previous studies evidenced active diapirism and suggested active fluid venting (Geletti et al., 2008). Although semi-volatile and non-volatile aliphatic hydrocarbons (C10-C40) and PAHs did not appear particularly elevated in the investigated sites, the overall results of this study demonstrated the presence of a hydrocarbon anomaly associated with the pockmark field. In previous studies, pockmarks have been described to be periodically active, perhaps only showing measurable fluid flow during special external events, such as extreme low pressure, extreme low tides, storm surges, or during increased fluid pressure (Hovland et al., 2002, 2010; Pilcher and Argent, 2007). The overall distribution of hydrocarbons concentrations along a SE-NW axis follows the alignment of the sampled pockmarks with the Middle Adriatic Ridge. Overall, these observations indicate that the pockmarks located in the proximity of the Middle Adriatic Ridge are associated with a sedimentary hydrocarbon anomaly and suggest that these sites are active, or have been active in the recent past.

The organic matter content of the pockmark sediments was comparable to deep-sea stations sampled previously in the Mediterranean Sea (Giovannelli et al., 2013b), and ca. 3 times lower that those measured in Adriatic coastal stations (Molari et al., 2012). Higher value of BPC and proteins were measured in SE pockmarks A162, A163, and A164 compared to station A168 and surrounding sediments A169 (Figure 3). The same SE-NW decreasing trend was observed for the CPE. Since all stations were in close proximity (within a 4 km radius) and were at similar depths, we assumed similar input of organic carbon from the water column. This implies that the measured differences in organic matter quantity and quality may be due to local processes and may reflect a complex interplay between biotic and abiotic factors over time (Hedges and Keil, 1995; Wakeham and Canuel, 2006). Measured nitrate+nitrite and ammonia concentrations differed in their downcore profiles between stations, with faster depletion of nitrate+nitrite in stations A164 and A168. All together our results indicate that pockmarks clustering in close proximity to each other may still diverge in terms of chemistry and sedimentary organic matter content, possibly connected to different stages of activity and successional status previously described for mud volcanoes (Neurauter and Roberts, 1994).

Higher benthic diversity has been associated with pockmark structures in the Mediterranean Sea (Zeppilli et al., 2012; Taviani et al., 2013; Sandulli et al., 2015), in the Atlantic Ocean (Olu-Le Roy et al., 2007), and North Sea (Dando et al., 1991; Dando, 2001), as well as at several other locations (Judd and Hovland, 2007). In pockmarks, spatially heterogeneous microbial and faunal assemblages have been reported in the past (Dando et al., 1991; Levin, 2005), possibly connected to differences in fluid regimes among pockmarks situated in the same fields. Prokaryotic abundances measured in this study were on average in line with other environments at comparable depth. There was a clear decrease in microbial biomass as the depth of the sediment layers increased, as previously described in other studies in the Mediterranean Sea (Corinaldesi et al., 2011; Molari et al., 2012; Giovannelli et al., 2013a). Prokaryotic biomass was comparable to other environments at similar depth, also when using more conservative estimates of the per cell carbon content. Generally, the decrease of microbial cell counts along the sediment vertical profile is the result of decreasing organic carbon quality and availability in aged buried sediments (Parkes et al., 2000). However, other abiotic and biotic factors have to be taken into account (Molari et al., 2012). In our studies the measured differences in nutrient and major cations concentrations did not explain the observed trends. The differences in prokaryotic abundances and biomass between the sampled stations correlates with higher hydrocarbon and sedimentary matter concentrations within the pockmarks, indicating a possible role of fluid circulation in controlling prokaryotic distribution. As previously described for the hydrocarbon content (especially for the volatile hydrocarbons C5-C10) and the sedimentary organic matter, the prokaryotic abundance and biomass were significantly higher at pockmarks A162, A163, and A164 (Figure 3), suggesting a possible higher prokaryotic activity at those stations. Increased prokaryotic counts associated with active seeping (Werne et al., 2004; Pimenov et al., 2010) and mud volcanoes (Mills et al., 2003; Corinaldesi et al., 2011) have been already reported in other areas worldwide. Conversely, lower bacterial abundances in inactive pockmarks compared to surrounding sediments were reported in the North Sea (Haverkamp et al., 2014).

Previous investigation in seep-influenced sediments suggested that a high proportion of *Archaea* is present in these environments, especially in subsurface sediment layers (Orcutt et al., 2005; Corinaldesi et al., 2011). This increase in *Archaea* abundance may be due to the higher presence of methanotrophs, especially linked to the anaerobic oxidation of methane (Joye et al., 2004; Orcutt et al., 2005; Knittel and Boetius, 2009). Although estimate of *Bacteria* and *Archaea* relative contributions are influenced by the methodology selected (Xie et al., 2013), the general increase in *Archaea* with sediment depths in the first few meters below seafloor is generally described also in non-seep sediments (Lipp et al., 2008; Molari et al., 2012). Our data suggest a different trend for the shallow-water pockmarks sampled in this study. The community was dominated by *Bacteria* in all sampled stations, and, except for station A162 in the deep layer, BAR increased with increasing sediment depth. This observation, along with the increase in hydrocarbon concentration in the deeper sediment layers, suggests a role for the bacterial population in hydrocarbon degradation, potentially linked to the use of oxidized nitrogen species.

We investigated the microbial diversity in the surface sediments (0–1 cm layer) using 16S rRNA pyrotag libraries. Our analysis revealed that the total diversity in the pockmarks was higher than that of the surrounding sediments (Figure 4). The overall diversity estimates (average Chao1 of 2873) were in line with those reported by Ruff et al. (2015) in their global estimate of diversity in cold seep-associated sediments. However, the composition of the microbial communities associated with the pockmarks of the Adriatic Sea differed from previously studied cold-seeps and pockmarks worldwide.

In order to investigate whether the different assemblages were dominated by few or several phylotypes, we calculated the core microbiome for the sampled stations (i.e., the OTUs present in all sampled stations), common OTUs (i.e., shared among some stations) and station-specific OTUs (Figure 7). We used the obtained information to draw the “connectome” (i.e., the network of connection among shared OTUs in our dataset; Figure 5, circle e), visually representing the distribution of the OTUs network among the different samples. The visual analysis of the connectome can reveal the presence of pattern among the distribution of OTUs between stations. For instance, the presence of asymmetrical connection would indicate that certain OTUs are common only among some stations. Our results show that: (i) there is a high degree of symmetry of the connectome, implying that on average the shared OTUs are ubiquitous among all sampled stations; and (ii) the majority of the station specific OTUs are part of the unclassified sequences pool, leaving little margin for interpretation of their possible ecological role.

The obtained libraries were all dominated by sequences related to the phylum *Firmicutes*. Members of this group have been previously found to be abundant in other hydrocarbon enriched sediments and soils (Vivas et al., 2008; Reda, 2009; Kostka et al., 2011; Lamendella et al., 2014), and are commonly isolated when enriching for facultative anaerobic hydrocarbon degraders (Yakimov et al., 2007; Meckenstock and Mouttaki, 2011; Scherr et al., 2012; Lamendella et al., 2014; Abbasian et al., 2015). Further, high relative contribution of sequences assigned to the phylum *Firmicutes* were previously reported for the alkaline, athalassohaline lakes of the Wadi An Natrun valley in Egypt (Mesbah et al., 2007), the Gulf of Mexico and Cascadia Margin gas hydrate associated sediments (Lanoil et al., 2001; Knittel et al., 2003), in terrestrial mud volcanoes (Chang et al., 2012) and Arctic sediments (Hubert et al., 2009) in proximity of pockmark features. The majority of OTUs in the *Firmicute*s phylum were associated with the order *Bacillale*s. The closest cultured relatives to the phylotypes retrieved from the pockmarks have heterotrophic metabolism, and most are known aliphatic or aromatic hydrocarbon utilizers. The most abundant OTU within the *Bacillale*s was related to the *B. aquimari*s, known to metabolize a wide variety of organic compounds and amino acids and recently shown to grow on hydrocarbons as its sole carbon and energy source (Babatunde, 2012; Syakti et al., 2013; Fathepure, 2014). While *Firmicutes* are generally involved in the degradation and fermentation of complex organic substrates, these recent data indicate that members of this phylum are able to degrade hydrocarbons. Sequences related to the *Actinobacteria* (high G+C division gram positive) were also retrieved in all investigated libraries (3.6% on average). Members of this division are also often retrieved from hydrocarbon-contaminated areas, and constituted up to 40% of the clones from gas hydrate from the Nankai Trough (Colwell et al., 2004).

OTUs related to the *Alteromonadales, Oceanospirillales*, and *Pseudomonadales*, within the class *Gammaproteobacteria*, occurred frequently in the pockmarks. Members of the *Alteromonadales* have been shown to be primary players in the aerobic degradation of hydrocarbons (Rosenberg, 2013), and are reported as primary responders in studies that assessed the microbial community following the 2010 oil spill in the Gulf of Mexico (Joye et al., 2014; Kleindienst et al., 2016). Other groups of *Gammaproteobacteria* potentially involved in the degradation of hydrocarbon were *Colwelliaceae, Pseudoalteromonadaceae, Oceanospirillaceae*, and *Alcanivoracaceae* (Kostka et al., 2011; Rosenberg, 2013; Joye et al., 2014; Kleindienst et al., 2016), although their relative abundance was lower. These groups have been described as abundant in water samples collected in hydrocarbon-contaminated areas (Kostka et al., 2011; Dubinsky et al., 2013).

A high number of sequences related to known thiotrophic and methylotrophic bacteria were recovered, belonging to the *Thiotrichales* and *Chromatiales* divisions in the *Gammaproteobacteria, Methylacidiphilae* in the *Verrucomicrobia*, and the *Methylarcula* in the *Alphaproteobacteria* division. Sequences relative to the order *Chromatiales* have been previously identified in deeper sediments of inactive pockmarks in the North Sea (Haverkamp et al., 2014). We also identified a high percentage of sequences belonging to the candidate division NC10, albeit with a low similarity to the only known cultured relative, *candidatus* Methylomirabilis oxyfera. Members of this division have been identified in low abundance in a variety of aquatic habitats worldwide and are now believed to be responsible for the anaerobic oxidation of methane coupled to denitrification, using a new “intra-aerobic” pathway (Raghoebarsing et al., 2006; Knittel and Boetius, 2009; Ettwig et al., 2010). The presence of sequences related to candidate division NC10 in our samples suggests that nitrite-dependent anaerobic methane and hydrocarbon oxidation might be a mechanism of hydrocarbon utilization in the pockmarks. Similar reactions may be coupled to more recalcitrant substrates (i.e., aromatic compounds and alkanes) (Ettwig et al., 2010).

We also observed the presence of numerous phylotypes related to anaerobic/microaerophilic bacteria in our libraries. Aside from possible anaerobic methane or hydrocarbon degraders, we detected sequences associated with known sulfate reducers. Several phylotypes were associated with sequences closely related to members of the *Mixococcales*, candidate division NB1-j, *Desulfuromonadaceae, Nitrospinaceae*, and *Geobacteraceae* divisions. Sequences related to the syntrophic sulfate reducers order *Syntrophobacterales* were also retrieved in the libraries. Members of these divisions are capable of sulfate reduction both as energy metabolism (chemolithotrophy) or associated to a fermentative type of metabolism (Barton and Tomei, 1995), and are known to play a fundamental role in sulfur cycling. The co-occurrence of sulfate reducers and thiotrophic groups, such as members of the order *Chromatiales*, indicate that a complete sulfur cycle may be present in pockmark sediments, highlighting the complementary interplay between the carbon and sulfur cycles in these environments.

Numerous retrieved phylotypes were also involved in the cycling of nitrogen species. Sequences related to the phylum *Nitrospirae*, the order *Nitrosomonadales* of the *Betaproteobacteria* divison and *Nitrospinaceae* family within the *Deltaproteobacteria* were retrieved all at relative abundances between 0.1 and 0.5%, suggesting potential for nitrite oxidation in the sampled sediments. Moreover, numerous phylotypes associated with the *Firmicutes, Gammaproteobacteria, Deltaproteobacteria*, and *Alphaproteobacteria* division are capable of respiring nitrate anaerobically. Sequences related to the *Planctomycetes*, representing the third most abundant bacterial phyla in our libraries, were potentially involved in nitrate reduction as well as anammox processes. Of the retrieved sequences belonging to the *Planctomycetes* pluylum, on average 1% was related to the *Kueneniae* class. Members of this group have been shown to be major players in the nitrogen cycle (Strous et al., 1999; Jetten et al., 2003), performing the anaerobic oxidation of ammonia. Cultured members of the *Planctomycea*, abundant in our libraries, include aerobic or facultative anaerobic chemoheterotrophs, some capable of nitrate reduction (Fuerst, 1995). The *Planctomycetes* phylum harbored the highest relative diversity in our libraries (Figure 6A), and was phylogenetically distant from cultured relatives. Similar results were previously reported for methane hydrate bearing sediments of the Nankai Trough (Reed et al., 2002), were the *Planctomycetes* represented the most diverse group identified in the study.

Overall, the presence of phylotypes related to anaerobic/microaerophilic bacteria in the 0–1 cm sediment layer of the pockmark sediments suggests a shallow penetration of oxygen. However, the presence of anaerobic niches in the surficial sediments may appear controversial. Under normal conditions, oxygen in finely grained marine sediments can diffuse up to several centimeters (Cai and Sayles, 1996; Glud, 2008). However, if high concentrations of organic carbon are present, like in the shallow-water sediments of the central Adriatic Sea, suboxic condition can be found within the first 10 mm (Yücel, 2013). Moreover, the effective penetration of oxygen is also influenced by the presence of upward fluid and gas venting (Yücel, 2013), and can be limited to the first few millimeters in cold-seeps and mud volcanoes (Niemann et al., 2006).

*Archaea*-related sequences were mostly represented by the phylum *Thaumarchaeota*, with members of the genus *Nitrosopumilus* being the most abundant. Previous studies showed that members of the *Thaumarchaeota* phylum drive CO_2_ fixation in deep waters coupled to ammonia oxidation (e.g., Könneke et al., 2005). The high abundance of sequences related to the *Thaumarchaeota* in all investigated libraries suggest an increased importance of carbon fixation linked to ammonia oxidation. Recently, sequences related to the *Thaumarchaeota* have been reported from diverse environments, including marine sediments (Auguet et al., 2009; Durbin and Teske, 2010) and basalts (Mason et al., 2008). We also identified sequences related to members of the *Euryarchaeota*, albeit at very low relative abundances. Sequences belonging to the class *Themoplasmata, Methanobacteria*, and *Halobacteria* occurred in very low abundances (i.e., below 0.1%), while ANME groups were completely absent. Previous studies carried out in methane-rich sediments, pockmarks and mud-volcanoes showed a high abundance of *Euryarchaeot*a associated with anaerobic methane oxidizers consortium (ANME) (Boetius et al., 2000; Joye et al., 2004; Kniemeyer et al., 2007), which couples sulfate-reduction to the anaerobic oxidation of methane in seep environments (reviewed in Knittel and Boetius, 2009). Members of the ANME divisions are strict anaerobes and they are usually present in deeper sediments in the sulfate-methane transition zone, where sulfate-reduction and methane oxidation co-occur (Knittel and Boetius, 2009).

**Figure 8:**
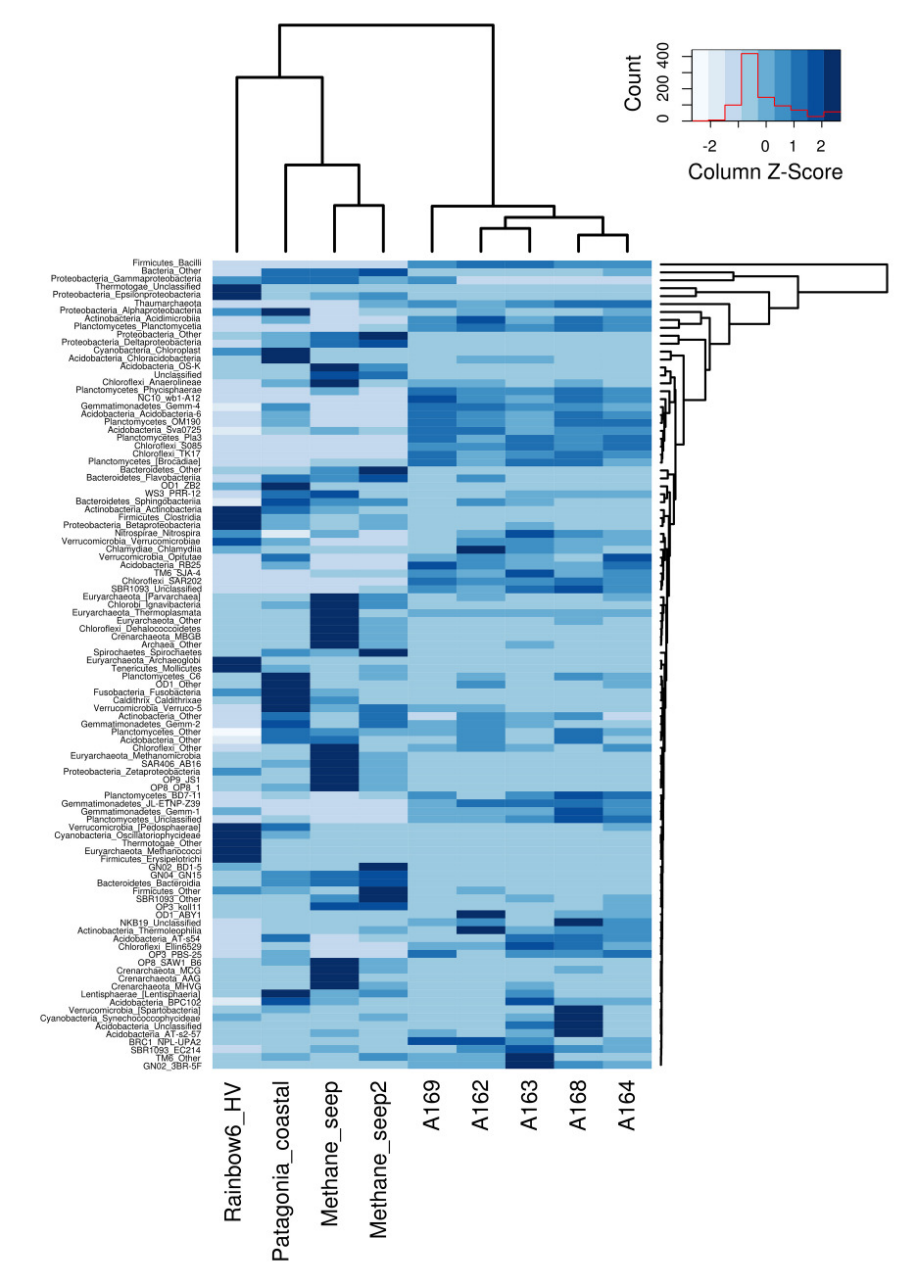
**Comparative 16S rRNA gene heatmap of diversity (at the class level) of shallow-water pockmark libraries (this study) against other 16S rRNA dataset publicly available**.

*Proteobacteria* and *Planctomycetes* had the highest number of station-specific OTUs in pockmark station A162, A163, and A164 (visible in the higher number of gray bands in Figure 5, circle b). This result implies that a specialized community is present in shallow-water pockmark sediments, and that shallow-water pockmarks harbor a prokaryotic community with a unique diversity signature. When compared to publicly available datasets from coastal waters, deep-sea hydrothermal vents, and deep-sea methane seeps, the sampled shallow-water pockmarks reveal a unique diversity pattern (Figure 8). Despite numerous phyla were shared among samples, the phylotypes associated with each environment were different, and pockmark libraries' obtained in this study presented unique groups not commonly found in the comparison libraries. The high abundance of unknown and unclassified sequences in pockmark stations suggest the presence of a considerable amount of microbial novelty, warranting further investigations, perhaps including culture-based approaches.

In conclusion, our results indicate that the pockmarks located in the proximity of the Middle Adriatic Ridge are associated with a sedimentary hydrocarbon anomaly, suggesting that these sites are active, or have been active in the recent past. Microbiological analyses indicate that the community associated with pockmark sediments is more diverse and harbors higher abundances than those in surrounding sediments, potentially due to the higher availability of organic matter and hydrocarbon concentrations linked to active seeping. The diversity signature of the shallow-water pockmarks of the Adriatic Sea appears to be unique and comprised phylotypes related to bacteria associated with the cycling of sulfur and nitrate compounds, as well as numerous known hydrocarbon degraders. Altogether, this study suggests that shallow-water pockmarks positively influence the benthic diversity, providing specialized environmental niches harboring a wide range of metabolically diverse prokaryotes, warranting further interest in their investigation.

## Author contributions

DG, FR, MT, and EM designed the research. DG, LA, MY, TB, and MT performed the sampling. DG, GD, FF, LA, DF, MY, and EM performed laboratory analyses. All authors contributed to the analysis and interpretation of the results and wrote the manuscript.

## Funding

This work was partially supported by a Postdoctoral Fellowship from the Center for Dark Energy Biosphere Investigations [C-DEBI, grant OCE-0939564] awarded to DG, by National Science Foundation grant OCE 11-24141 to CV, and European Science Foundation EuroDeep BIOFUN grant CTM2007-28739-E to EM. This article commits to EU HERMIONE [contract no. 226354] and CoCoNet [contract no. 287844] programs, and the Italian MIUR flag Ritmare within the National Research Program 2011–2013. This publication was in part supported by the ELSI Origins Network (EON), which is supported by a grant from the John Templeton Foundation. The opinions expressed in this publication are those of the authors and do not necessarily reflect the views of the John Templeton Foundation. Porewater chemical measurements were supported by DEKOSIM Project (National Excellence Centre for Marine Ecosystem and Climate Research - Deniz Ekosistem ve İklim Araştırmaları Merkezi, Project Code BAP-08-11-DPT.2012K120880), financed by the Ministry of Development of Turkey. This is ISMAR-CNR, Bologna scientific contribution n. 1842.

### Conflict of interest statement

The authors declare that the research was conducted in the absence of any commercial or financial relationships that could be construed as a potential conflict of interest.
